# Social mobility and healthy behaviours from a gender perspective in the Spanish multicase-control study (MCC-Spain)

**DOI:** 10.1371/journal.pone.0251447

**Published:** 2021-05-12

**Authors:** M. Pinto-Carbó, R. Peiró-Pérez, A. Molina-Barceló, M. Vanaclocha-Espi, J. Alguacil, G. Castaño-Vinyals, C. O’Callaghan-Gordo, E. Gràcia-Lavedan, B. Pérez-Gómez, V. Lope, N. Aragonés, A. J. Molina, T. Fernández-Villa, L. Gil-Majuelo, P. Amiano, T. Dierssen-Sotos, I. Gómez-Acebo, M. Guevara, C. Moreno-Iribas, M. Obón-Santacana, M. M. Rodríguez-Suárez, I. Salcedo-Bellido, A. Delgado-Parrilla, R. Marcos-Gragera, M. D. Chirlaque, M. Kogevinas, M. Pollán, D. Salas

**Affiliations:** 1 Cancer and Public Health Area, Foundation for the Promotion of the Research in Healthcare and Biomedicine (FISABIO-Salud Pública), Valencia, Valencian Community, Spain; 2 Inequalities Area, Foundation for the Promotion of the Research in Healthcare and Biomedicine (FISABIO-Salud Pública), Valencia, Valencian Community, Spain; 3 General Directorate of Public Health, Valencia, Valencian Community, Spain; 4 Consortium for Biomedical Research in Epidemiology and Public Health (CIBEResp), Madrid, Spain; 5 Centre for Health and Environmental Research, Huelva University, Huelva, Andalucia, Spain; 6 Barcelona Institute for Global Health (ISGlobal), Barcelona, Catalonia, Spain; 7 Universitat Pompeu Fabra, Plaça de la Mercè, Barcelona, Catalonia, Spain; 8 Hospital del Mar Medical Research Institute, Barcelona, Catalonia, Spain; 9 Faculty of Health Science,Universitat Oberta de Catalunya, Barcelona, Catalonia, Spain; 10 Department of Epidemiology of Chronic Diseases, National Center for Epidemiology, Carlos III Institute of Health, Madrid, Spain; 11 Epidemiology Section, Public Health Division, Department of Health of Madrid, Madrid, Spain; 12 The Research Group in Gene—Environment and Health Interactions, Institute of Biomedicine (IBIOMED), University of León, León, Castilla y León, Spain; 13 Ministry of Health of the Basque Government, Sub-Directorate for Public Health and Addictions of Gipuzkoa, San Sebastián, Gipuzkoa, Spain; 14 Biodonostia Health Research Institute, Group of Epidemiology of Chronic and Communicable Diseases, San Sebastián, Gipuzkoa, Spain; 15 Cantabria University, Santander, Cantabria, Spain; 16 Navarra Public Health Institute, Pamplona, Navarra, Spain; 17 Navarra Institute for Health Research (IdiSNA), Pamplona, Navarra, Spain; 18 Oncology Data Analytics Program (ODAP), Catalan Institute of Oncology (ICO), Barcelona, Catalonia, Spain; 19 ONCOBELL Program, Bellvitge Biomedical Research Institute (IDIBELL), Barcelona, Catalonia, Spain; 20 Preventive Medicine and Public Health Area, Oviedo University, Oviedo, Asturias, Spain; 21 Central University Hospital of Asturias, Public Health Service of the Principe de Asturias, Oviedo, Asturias, Spain; 22 Instituto de Investigación Biosanitaria ibs.GRANADA, Granada, Andalucia, Spain; 23 Departamento de Medicina Preventiva y Salud Pública, Universidad de Granada, Granada, Andalucia, Spain; 24 Epidemiology Unit and Girona Cancer Registry, Oncology Coordination Plan, Department of Health, Autonomous Government of Catalonia, Catalan Institute of Oncology, Girona, Catalonia, Spain; 25 Descriptive Epidemiology, Genetics and Cancer Prevention Group [Girona Biomedical Research Institute], Girona, Catalonia, Spain; 26 Department of Epidemiology, Murcia Regional Health Council, IMIB-Arrixaca, El Palmar, Murcia, Spain; Ruhr University Bochum, GERMANY

## Abstract

There is evidence for the influence of socioeconomic status (SES) on healthy behaviours but the effect of social mobility (SM) is not yet well known. This study aims to analyse the influence of origin and destination SES (O-SES and D-SES) and SM on healthy behaviours and co-occurrence, from an integrated gender and age perspective. Data were obtained from the controls of MCC-Spain between 2008–2013 (3,606 participants). Healthy behaviours considered: healthy diet, moderate alcohol consumption, non-smoking and physical activity. SM was categorized as stable high, upward, stable medium, downward or stable low. Binary and multinomial logistic regression models were adjusted. Those aged <65, with a low O-SES, D-SES and stable low SM are less likely to have healthy behaviours in the case of both women (physically active: OR = 0.65 CI = 0.45–0.94, OR = 0.71 CI = 0.52–0.98, OR = 0.61 CI = 0.41–0.91) and men (non-smokers: OR = 0.44 CI = 0.26–0.76, OR = 0.54 CI = 0.35–0.83, OR = 0.41 CI 0.24–0.72; physically active: OR = 0.57 CI = 0.35–0.92, OR = 0.64 CI = 0.44–0.95, OR = 0.53 CI = 0.23–0.87). However, for those aged ≥65, this probability is higher in women with a low O-SES and D-SES (non-smoker: OR = 8.09 CI = 4.18–15.67, OR = 4.14 CI = 2.28–7.52; moderate alcohol consumption: OR = 3.00 CI = 1.45–6.24, OR = 2.83 CI = 1.49–5.37) and in men with a stable low SM (physically active: OR = 1.52 CI = 1.02–1.26). In the case of men, the same behaviour pattern is observed in those with a low O-SES as those with upward mobility, with a higher probability of co-occurring behaviours (three-to-four behaviours: OR = 2.00 CI = 1.22–3.29; OR = 3.13 CI = 1.31–7.48). The relationship of O-SES, D-SES and SM with healthy behaviours is complex and differs according to age and gender.

## Introduction

Social determinants of health are the conditions in which people are born, grow up, work and live that affect their values, preferences and access to resources, and therefore their health behaviours and health outcomes [1]. Thus, health behaviours are socially determined and result in health inequalities, with gender and socioeconomic status (SES) being key social determinants [2]. Some of the most important healthy behaviours for management of chronic diseases include a diet rich in fruits and vegetables, the absence of smoking, responsible alcohol consumption and physical activity [3].

It has been observed that populations with a lower SES have more unhealthy behaviours [4–8]. Gender inequalities in health-related behaviours have also been found, with alcohol consumption being the most common unhealthy behaviour in men and physical inactivity in women [9]. Moreover, taking into account that the worst health indicators are seen in women with a lower SES, it is important to explore how the intersection between these two axes of inequality also determines health behaviours [10].

Health behaviours usually appear cumulatively, with a co-occurrence of two or more risk behaviours being the most common scenario [5, 9]. The bault’s cross-sectional study concluded that, the accumulation of risk behaviours was similar between both genders although the greatest predisposition is observed in men with a low educational level [11]. Therefore, the intersection between gender and educational level is also important when studying the co-occurrence of health behaviours.

There are several studies that show the influence of SES on health behaviours and health outcomes [11, 12], but the effect of social mobility is not yet well known. Social mobility can be understood from an intragenerational perspective, meaning, the change in an individual’s SES throughout his or her lifecourse or from an intergenerational perspective, meaning, changes in SES between two generations (progenitors-children) [13]. This study is framed from an intergenerational social mobility perspective, since it considers the SES of the parents and the participants.

Campos-Matos’s study [14] shows that the upwardly mobile population, that is, those who ascend the social scale, present better health than the downwardly mobile population, that is, those who descend it. Regarding health behaviours, another study observed that people with stable low, meaning they remain in a low social position, or downward mobility are more likely to have unhealthy behaviours [5]. Gugushvili’s study [15] shows a differential mobility pattern according to gender, with women being more influenced by their origin SES than men. These findings also coincide with other studies [16]. Considering Van Eijck’s sociological theories regarding the impact of social mobility on behaviours [17], the results of aforementioned studies [15, 16] coincide with the socialization hypothesis, which puts forward that primary socialization has a stronger impact on healthy behaviours than secondary socialization. Therefore, our hypothesis is that origin SES influences health behaviours to a greater extent than destination SES, depending on gender and age, with different results according to the type of behaviour studied.

SES can be measured by income, occupation or educational level [18]. To analyse intergenerational social mobility, this study used the origin SES obtained through the self-referred position of the progenitors and the destination SES obtained through the current educational level of participants, used as an approximation of their status.

This study aims to analyse the influence of origin and destination SES and social mobility on healthy behaviours, as well as co-occurrence, from an integrated gender and age perspective.

## Materials and methods

### Design and study population

We performed a cross-sectional design using data from men and women recruited as population-based controls in the Multicase Control study in Spain (MCC-Spain). The MCC-Spain is a case-control study on several types of cancers, namely breast, prostate, colorectal, gastric and chronic lymphocytic leukemia, carried out in 10 Spanish regions (Madrid, Catalonia, Valencian Community, Andalucia, Navarra, Castilla León, Murcia, Asturias, Basque Country, and Cantabria). Its main characteristics have been described elsewhere [19]. For the present study, only population without cancer (controls) was included. Selected controls were recruited between September 2008 and December 2013, and were randomly selected from the administrative records of primary care health centres located within the catchment areas [19]. These controls were frequency-matched by age, sex and region, ensuring that in each region there was at least one control of the same sex and 5-year interval for each case. All controls had to be between 20–85 years of age, to have resided in the catchment area for at least 6 months before the selection and to be able to answer the epidemiological questionnaire [19].

The sample of this study consisted of 4,098 participants. Participants that were missing information on any of the study variables were excluded from this analysis (492 excluded). Therefore, the study sample included 3,606 participants aged between 24 and 85 (1,762 women and 1,844 men).

### Data collection

Information on sociodemographic characteristics, tobacco habits and physical activity was collected from the epidemiological MCC-Spain questionnaire, developed by MCC-Spain researchers. Food intake and alcohol consumption were evaluated using a self-administered, semi-quantitative food frequency questionnaire (FFQ) [20] validated in Spain to include regional products.

All these data were collected in a cross-sectional manner when the controls accepted participation in the study.

### Ethics statement

Participants who agreed to partake in the study signed an informed consent form. The MCC-Spain study protocol was approved by the Clinical Research Ethics Committee of the Municipal Health Care Institute (IMAS) in accordance with conformity to the principles of the Declaration of Helsinki. The database was registered with the Spanish data protection agency, under number 210267217118 [19].

### Study variables

#### Origin SES

We use the SES of progenitors, obtained through a self-referred question. This variable is categorized as low, medium or high.

#### Destination SES

The participants’ current educational level is used as an indicator of the destination SES [18]. This variable is categorized as low (less than or equal to primary school level), medium (secondary studies) or high (university studies).

#### Social mobility (intergenerational)

Social mobility was categorized into five groups, taking into account the origin and destination SES: 1) “stable high", which includes participants that have a high origin SES and high destination SES; 2) "stable medium", which includes participants that have a medium origin SES and medium destination SES; 3)"stable low", which includes participants that have a low origin SES and low destination SES; 4) "upward", which includes participants that have a low origin SES and medium/high destination SES, or that have medium origin SES and high destination SES; and 5) "downward", which includes participants that have a high origin SES but have medium/low destination SES, or that have a medium origin SES but now have low destination SES.

#### Healthy behaviours

We studied four healthy behaviours: 1) healthy diet, considered as the consumption of ≥400 g/d of fruits and vegetables [21]. Intake from previous years was evaluated.; 2) tobacco consumption, grouping the responses into smokers (former and current smokers) and non-smokers (never smoked before); 3) alcohol consumption, measured as the number of glasses consumed per day and categorized in Standard Drinking Units (SDU). According to the recommendations of the Dietary Guidelines for Americans [22], moderate consumption is considered as ≤one SDU/day for women and ≤ two SDU/day for men; 4) physical activity, evaluated using metabolic equivalents (METs), establishing adequate physical activity as a weekly energy expenditure in physical activities higher than 8 METs hour per week [23], that is to say, consuming 8 times the energy consumed at rest. Physical activity referred to a long period of time (approximately 10 years).

#### Co-occurrence of healthy behaviours

This refers to the coexistence of behaviours and was categorized into: none, one, two, three and four healthy behaviours. Three groups were established for the analysis: none-to-one, two and three-to-four. These categories allowed us to compare extreme groups, i.e., few healthy behaviours (none-to-one) versus many healthy behaviours (three-to-four).

### Statistical analysis

Information on age, origin and destination SES, social mobility, healthy behaviours and co-occurrence was obtained for the entire sample and expressed in frequencies and percentages.

Logistic regression models were used to study the association between origin and destination SES and social mobility (explanatory variables) for each of the healthy behaviours studied (outcome variables). These models were first calculated for total sample (adjusted by age, gender, interaction age*gender and region) and stratified by age and gender groups later on (adjusted by region). Age, gender and region were considered as confounding factors. The sample was stratified by gender as gender roles can lead to inequalities in health behaviours. The age stratification used two categories: under 65 and over 65. The population aged under 65 was born in the 50s, and therefore achieved social and economic independence around the 70s when political, social and economic changes took place in Spain towards greater democratization which improved gender and social equity. This social transformation may have reduced social and gender inequalities, and may also have influenced the health behaviour patterns of the population.

Multinomial logistic regression models were used to study the association between origin and destination SES and social mobility (explanatory variables) with the co-occurrence of healthy behaviours (outcome variables) (category of reference: none-to-one behaviours). All models were adjusted by region and stratified by gender and age to control their role as confounding factors. Results were presented in terms of the odds ratio and a 95% confidence interval. Statistical analysis was performed using SPSS Statistics V21.

## Results

In Table 1, the characteristics of study participants can be seen. In both genders, the most represented social mobility category is stable low, and the least represented category is upward. The most frequent healthy behaviour in women is moderate alcohol consumption and the least frequent is being physically active. In men, the most frequent is moderate alcohol consumption and the least frequent is being a non-smoker. Both genders are most likely to present co-occurrence of three-to-four behaviours (Table 1).

**Table 1:** Characteristics of the participants (2008–2013, Spain).

	Women		Men		Total	
	n	%	n	%	n	%
	1,762	48.9	1,844	51.1	3,606	100
**Age**						
<65	1,076	61.1	724	39.3	1,800	49.9
≥65	686	38.9	1,120	60.7	1,806	50.1
**Geographical area**						
Madrid	324	18.4	318	17.2	642	17.8
Catalonia	404	22.9	586	31.8	990	27.5
Valencian Community	52	3.0	57	3.1	109	3.0
Andalucia	83	4.7	173	9.4	256	7.1
Navarra	158	9.0	81	4.4	239	6.6
León	204	11.6	235	12.7	439	12.2
Murcia	12	0.7	28	1.5	40	1.1
Asturias	113	6.4	102	5.5	215	6.0
Basque Country	250	14.2	94	5.1	344	9.5
Cantabria	162	9.2	170	9.2	332	9.2
**Origin SES**						
High	302	17.7	282	16.2	584	16.9
Medium	873	51.1	868	49.7	1,741	50.4
Low	534	31.2	595	34.1	1,129	32.7
**Destination SES**						
High	379	21.5	393	21.3	772	21.4
Medium	545	30.9	511	27.7	1,056	29.3
Low	838	47.6	940	51.0	1,778	49.3
**Social mobility**						
Stable high	278	16.3	270	15.5	584	15.9
Upward	113	6.6	119	6.8	232	6.7
Stable medium	500	29.2	474	27.2	974	28.2
Downward	297	17.4	292	16.7	589	17.0
Stable low	521	30.5	590	33.8	1,111	32.2
Healthy eating* **(Yes)**	1,243	70.5	1,130	61.3	2,373	65.8
Non-smoker* **(Yes)**	1,072	60.8	527	28.6	1,599	44.3
Moderate alcohol consumption*(Yes)	1,478	83.9	1,215	65.9	2,693	74.7
Physically active* **(Yes)**	809	45.9	1,007	54.6	1,816	50.4
Co. Healthy behaviours*						
None-to-one	247	14.0	533	28.9	780	21.6
Two	523	29.7	634	34.4	1157	32.1
Three-to-four	992	56.3	677	36.7	1669	46.3

* The table shows the percentage of participants that practised each behaviour, out of the total number of participants.

In the logistic regression models for the entire sample (Table 2) it is observed that the population with low origin and destination SES (OR = 1.23 CI = 1.01–1.50, OR = 1.40 CI = 1.11–1.76), as well as those with stable medium mobility (OR = 1.38 CI = 1.09–1.75), are more likely to be non-smoker. In addition, this table shows that, regardless of origin and destination SES and social mobility, sex influences all healthy behaviors (p <0.05), that age in turn impacts healthy diet and physical activity and finally, that the interaction between sex and age has an impact on be non-smoker.

**Table 2:** Logistic regression models for each of the healthy behaviours and interaction tests with age and sex for the whole sample (2008–2013, Spain).

	Healthy diet		Non-smoker		Moderate alcohol consumption		Physically active	
	OR	CI (95%)	OR	CI (95%)	OR	CI (95%)	OR	CI (95%)
**Origin SES**								
High	1		1		1		1	
Medium	0.93	0.76–1.14	0.88	0.72–1.08	0.91	0.72–1.13	0.84	0.70–1.00
Low	1.05	0.86–1.27	1.23	1.01–1.50*	1.00	0.81–1.24	0.90	0.75–1.09
**Sex**								
Men	1		1		1		1	
Women	1.89	1.50–2.32*	2.47	2.00–3.04*	9.94	7.85–12.57*	0.74	0.61–0.89*
**Age** (years)								
<65	1		1		1		1	
≥65	1.75	1.40–2.14*	1.01	0.82–1.26	0.85	0.69–1.03	1.49	1.23–1.81*
**Interaction sex-age**	0.96	0.71–1.29	4.89	3.57–6.72*	1.27	0.89–1.79	0.97	0.74–1.28
**Destination SES**								
High	1		1		1		1	
Medium	0.91	0.74–1.11	0.99	0.81–1.22	0.88	0.70–1.11	0.89	0.74–1.08
Low	1.13	0.90–1.42	1.40	1.11–1.76*	1.00	0.77–1.28	0.88	0.71–1.08
**Sex**								
Men	1		1		1		1	
Women	1.93	1.57–2.37*	2.39	1.93–2.95*	9.99	7.87–12.69*	0.72	0.59–0.88*
**Age** (years)								
<65	1		1		1		1	
≥65	1.75	1.42–2.14*	1.05	0.84–1.30	0.83	0.67–1.01	1.41	1.16–1.72*
**Interaction sex-age**	0.90	0.66–1.22	4.69	3.40–6.47*	1.29	0.91–1.84	1.01	0.76–1.34
**Social mobility**								
Stable high	1		1		1		1	
Upward	0.88	0.64–1.22	1.12	0.80–1.56	0.82	0.57–1.19	1.12	0.82–1.54
Stable medium	1.13	0.90–1.44	1.38	1.09–1.75*	0.97	0.75–1.25	0.87	0.70–1.08
Downward	0.88	0.68–1.14	1.10	0.85–1.43	0.88	0.66–1.17	0.88	0.69–1.13
Stable low	0.91	0.73–1.14	0.89	0.71–1.13	0.85	0.66–1.10	0.89	0.72–1.10
**Sex**								
Men	1		1		1		1	
Women	1.93	1.57–2.37*	2.40	1.94–2.97*	10.0	7.88–12.71*	0.72	0.59–0.88*
**Age** (years)								
<65	1		1		1		1	
≥65	1.74	1.42–2.14*	1.04	0.84–1.29	0.82	0.67–1.01	1.43	1.17–1.75*
**Interaction sex-age**	0.90	0.66–1.22	4.64	3.36–6.39*	1.29	0.91–1.84	1.02	0.77–1.35

*Statistically significant results (p<0.05).

Logistic regression model adjusted by region, sex, age and interaction sex-age.

Stratifying by sex and age, as detailed in Table 3, women under 65 with a low origin and destination SES and stable low mobility are more likely to be non-smokers (OR = 1.83 CI = 1.26–2.66, OR = 1.63 CI = 1.18–2.24, OR = 1.69 CI = 1.14–2.51) and less likely to be physically active (OR = 0.65 CI = 0.45–0.94, OR = 0.71 CI = 0.52–0.98, OR = 0.61 CI = 0.41–0.91). Women over 65 with a low origin and destination SES are more likely to be non-smokers (OR = 8.09 CI = 4.18–15.67, OR = 4.14 CI = 2.28–7.52) and those with a low origin and destination SES and stable low mobility consume moderate amounts of alcohol (OR = 3.00 CI = 1.45–6.24, OR = 2.83 CI = 1.49–5.37 OR = 3.97 CI = 1.81–8.70).

**Table 3:** Logistic regression models for each of the healthy behaviours stratified by gender and age (2008–2013, Spain).

Aged under 65								
	Healthy diet		Non-smoker		Moderate alcohol consumption		Physically active	
	OR	CI (95%)	OR	CI (95%)	OR	CI (95%)	OR	CI (95%)
**Women, origin SES**								
High	1		1		1		1	
Medium	0.97	0.71–1.32	1.06	0.79–1.43	1.24	0.82–1.87	0.72	0.54–0.98*
Low	1.38	0.93–2.06	1.83	1.26–2.66*	1.73	0.99–3.02	0.65	0.45–0.94*
**Women, destination SES**								
High	1		1		1		1	
Medium	0.98	0.72–1.34	0.98	0.73–1.33	0.94	0.65–1.38	0.70	0.52–0.95*
Low	1.00	0.72–1.40	1.63	1.18–2.24*	1.16	0.76–1.77	0.71	0.52–0.98*
**Women, social mobility**								
Stable high	1		1		1		1	
Upward	0.78	0.48–1.29	1.18	0.73–1.90	1.17	0.63–2.17	0.88	0.54–1.43
Stable medium	0.96	0.68–1.37	0.95	0.68–1.33	1,02	0.68–1.55	0.64	0.45–0.89*
Downward	0.67	0.42–1.04	1.45	0.94–2.23	1.22	0.69–2.16	0.84	0.55–1.30
Stable low	1.23	0.80–1.88	1.69	1.14–2.51*	1.28	0.76–2.16	0.61	0.41–0.91*
**Men, origin SES**								
High	1		1		1		1	
Medium	0.81	0.53–1.23	0.61	0.40–0.94*	0.51	0.33–0.76*	0.66	0.43–0.99*
Low	1.00	0.60–1.62	0.44	0.26–0.76*	0.33	0.20–0.55*	0.57	0.35–0.92*
**Men, destination SES**								
High	1		1		1		1	
Medium	0.98	0.72–1.34	0.55	0.36–0.84*	1.05	0.71–1.57	0.76	0.51–1.11
Low	1.00	0.72–1.40	0.54	0.35–0.83*	0.97	0.65–1.44	0.64	0.44–0.95*
**Men, social mobility**								
Stable high	1		1		1		1	
Upward	0.66	0.36–1.21	0.66	0.34–1.25	0.35	0.19–0.66*	0.70	0.38–1.28
Stable medium	0.74	0.47–1.17	0.51	0.32–0.82*	0.68	0.42–1.11	0.64	0.41–1.00
Downward	1.43	0.81–2.53	0.65	0.36–1,15	0.68	0.38–1.22	0.62	0.36–1.08
Stable low	1.10	0.67–1.82	0.41	0.24–0.72*	0.82	0.48–1.40	0.53	0,23–0,87*
**Aged 65 or older**								
	**Healthy diet**		**Non-smoker**		**Moderate alcohol consumption**		**Physically active**	
	OR	CI (95%)	OR	CI (95%)	OR	CI (95%)	OR	CI (95%)
**Women, origin SES**								
High	1		1		1		1	
Medium	0.66	0.32–1.34	2.19	1.21–3.96*	1.48	0.74–2.97	1.23	0.70–2.17
Low	0.86	0.42–1.75	8.09	4.18–15.67*	3.00	1.45–6.24*	1.14	0.65–1.99
**Women, destination SES**								
High	1		1		1		1	
Medium	0.89	0.41–1.95	1.43	0.74–2.78	1.61	0.77–3.38	1.83	0.98–3.43
Low	0.69	0.35–1.37	4.14	2.28–7.52*	2.83	1.49–5.37*	1.26	0.74–2.17
**Women, social mobility**								
Stable high	1		1		1		1	
Upward	0.74	0.16–3.32	0.99	0.28–3.59	0,93	0,24–3,56	0,83	0,25–2,80
Stable medium	0.83	0.35–1.98	4.22	2.30–7.77*	2,00	0,87–4,62	1,71	0,86–3,41
Downward	0.46	0.20–1.06	1.22	0.68–2.20	1,38	0,64–2,97	1,23	0,64–2,38
Stable low	0.73	0.33–1.61	1.55	0.27–1.15	3,97	1,81–8,70*	1,28	0,68–2,39
**Men, origin SES**								
High	1		1		1		1	
Medium	1.13	0.77–1.65	1.06	0.71–1.60	1.05	0.72–1.55	1.33	0.92–1.91
Low	1.46	0.99–2.16	1.32	0.87–1.98	1.30	0.89–1.92	1.41	0.97–2.04
**Men, destination SES**								
High	1		1		1		1	
Medium	0.96	0.64–1.44	0.97	0.64–1.49	1.00	0.67–1.49	1.18	0.80–1.73
Low	1.13	0.79–1.61	1.03	0.71–1.48	1.08	0.77–1.53	1.11	0.80–1.56
**Men, social mobility**								
Stable high	1		1		1		1	
Upward	1.60	0.77–3.29	1.27	0.65–2.47	1.29	0.64–2.60	2.60	1.28–5.29*
Stable medium	1.12	0.72–1.75	1.20	0.84–1.71	1.09	0.70–1.70	1.45	0.94–2.22
Downward	1.20	0.75–1.92	1.04	0.68–1.59	1.03	0.65–1.62	1.24	0.79–1.94
Stable low	1.40	0.92–2.13	1.00	0.62–1.60	1.24	0.82–1.87	1.52	1.02–1.26*

*Statistically significant results (p<0.05).

All logistic regression models adjusted by region.

For men, those under 65 with a low origin and destination SES and stable low mobility are less likely to be non-smokers (OR = 0.44 CI = 0.26–0.76, OR = 0.54 CI = 0.35–0.83, OR = 0.41 CI = 0.24–0.72) and be physically active (OR = 0.57 CI = 0.35–0.92, OR = 0.64 CI = 0.44–0.95 OR = 0.53 CI = 0.23–0.87). Those with a low origin SES and upward mobility are less likely to consume moderate amounts of alcohol (OR = 0.33 CI = 0.20–0.55, OR = 0.35 CI = 0.19–0.66). Men over 65 with stable low mobility are more likely to be physically active (OR = 1.52 CI = 1.02–1.26) (Table 3).

On the other hand, as shown in the multinomial regression models (Table 4), women over 65 with a low origin and destination SES and stable low mobility are more likely to have three-to-four behaviours (OR = 6.54 CI = 2.38–17.97, OR = 3.92 CI = 1.69–9.09, OR = 6.19 CI = 2.09–18.34) vs. none-to-one than those with a high origin and destination SES and stable high mobility.

**Table 4:** Multinomial regression models with the reference category “none- to-one healthy behaviour” stratified by gender and age (2008–2013, Spain).

Aged under 65				
	Two behaviours		Three or four behaviours	
	OR	CI (95%)	OR	CI (95%)
**Women, origin SES**				
High	1		1	
Medium	0.69	0.45–1.06	0.70	0.46–1.07
Low	0.72	0.40–1.27	1.13	0.66–1.95
**Women, destination SES**				
High	1		1	
Medium	0.62	0.41–0.95*	0.69	0.45–1.04
Low	0.65	0.41–1.04	0.96	0.61–1.50
**Women, social mobility**				
Stable high	1		1	
Upward	1.20	0.59–2.44	0.96	0.47–1.95
Stable medium	0.63	0.40–1.02	0.64	0.40–1.02
Downward	0.61	0.33–1.12	0.70	0.39–1.26
Stable low	0.69	0.38–1.23	0.99	0.61–1.89
**Men, origin SES**				
High	1		1	
Medium	0.61	0.36–1.04	0.33	0.20–0.57*
Low	0.65	0.35–1.19	0.36	0.19–0.66*
**Men, destination SES**				
High	1		1	
Medium	0.71	0.45–1.13	0.50	0.31–0.79*
Low	1.04	0.65–1.66	0.63	0.39–1.66
**Men, social mobility**				
Stable high	1		1	
Upward	0.50	0.24–1.05	0.27	0.13–0.59*
Stable medium	0.57	0.32–1.00	0.32	0.18–0.56*
Downward	0.97	0.48–1.93	0.53	0.26–1.05
Stable low	0.68	0.37–1.27	0.39	0.21–0.73*
**Aged 65 or older**				
	**Two behaviours**		**Three or four behaviours**	
	OR	CI (95%)	OR	CI (95%)
**Women, origin SES**				
High	1		1	
Medium	1.35	0.52–3.49	1.91	0.79–4.60
Low	3.09	1.05–9.06*	6.54	2.38–17.97*
**Women, destination SES**				
High	1		1	
Medium	1.94	0.65–5.84	3.00	1.08–8.27*
Low	2.17	0.87–5.34	3.92	1.69–9.09*
**Women, social mobility**				
Stable high	1		1	
Upward	0.42	0.06–2.75	0.58	0.11–3.00
Stable medium	1.90	0.55–6.59	3.27	1.02–10.48*
Downward	0.95	0.32–2.81	1.31	0.48–3.60
Stable low	2.49	0.88–8.81	6.19	2.09–18.34*
**Men, origin SES**				
High	1		1	
Medium	0.99	0.63–1.57	1.54	0.95–2.49
Low	1.03	0.63–1.67	2.00	1.22–3.29*
**Men, destination SES**				
High	1		1	
Medium	0.82	0.50–1.32	1.09	0.68–1.75
Low	1.04	0.69–1.59	1.24	0.82–1.89
**Men, social mobility**				
Stable high	1		1	
Upward	1.08	0.42–2.77	3.13	1.31–7.48*
Stable medium	0.80	0.48–1.35	1.49	0.87–2.54
Downward	1.20	0.70–2.05	1.37	0.77–2.43
Stable low	1.04	0.64–1.69	2.04	1.23–3.39*

*Statistically significant results.

Logistic regression model adjusted by region.

For men, those under 65 with a low origin SES and upward or stable low mobility are less likely to have three-to-four behaviours (OR = 0.36 CI = 0.19–0.66, OR = 0.27 CI = 0.13–0.59, OR = 0.39 CI = 0.21–0.73) vs. none-to-one than those with a high origin SES and stable high mobility. Men over 65 with a low origin SES and upward or stable low mobility are more likely to have three-to-four healthy behaviours (OR = 2.00 CI = 1.22–3.29, OR = 3.13 CI = 1.31–7.48, OR = 2.04 CI = 1.23–3.39).

## Discussion

This study shows that there are gender and age inequalities in terms of the impact of origin and destination SES and social mobility on the prevalence and co-occurrence of healthy behaviours, with different results depending on the type of behaviour. Those aged under 65 are less likely to have healthy behaviours if they have stable low social mobility, and the opposite is true in women and men over 65. Moreover, the same age trend was observed in the co-occurrence of healthy behaviours in men with a low origin SES and those with upward social mobility.

Some studies have observed that the absence of tobacco use and moderate alcohol consumption occur more frequently in women than men [24, 25]. From a gender perspective, these inequalities can be interpreted by the fact that, in traditional societies with a clear gender division of labour, both behaviours were only socially accepted in the productive, public and power spheres, which were historically occupied by men [26, 27]. The results of this study are in line with these studies [24, 25] and could indicate that traditional gender stereotypes are still evident in the population.

In this study, it is observed that women with low SES, regardless of their origin and destination, show healthy smoking and alcohol consumption behaviours. This is in line with others studies [27]. These results can also be explained by the influence of traditional gender roles, which are mainly present in women from low social groups. In addition, this connection is stronger in older women and could be due to the social changes that occurred in Spain during the 70s. In this decade, women were gradually incorporated into education and the labour market, which reduced socioeconomic and gender inequalities not only between men and women, but also between women themselves. As a consequence of this social transformation, women acquired behaviours associated with the traditional male role, such as the consumption of tobacco and alcohol, and social and gender inequalities in health behaviours reduced [28]. However, this increased equity may have had a lower impact on the older women in our sample, who seems to maintain traditional gender roles.

In contrast to women, some studies show that younger men with low SES are less likely to be non-smokers [29, 30], in line with our results. Traditionally speaking, tobacco consumption was associated with male behaviour as a symbol of power. However, this stereotype began to change in the 80s due to public health policies against this unhealthy behaviour [31]. From this point, the number of non-smokers gradually increased, but consumption remained unchanged in men with a low SES.

It should be noted that this study shows the same trend of healthy behaviour in relation to alcohol in both younger men with low origin SES and those with upward mobility. Taking this into account, we could suppose that men with upward mobility maintain the behaviours of the origin SES. This finding is in line with the socialization hypothesis, confirming the strong impact of primary socialization on men’s healthy behaviour patterns [17].

There is possibly a negative relationship between women with downward mobility and healthy diet, regardless of age. Bonaccio et al’s study [32] shows that the increase in the price of healthy food decreases access to these types of products, especially in the low-income population. Considering that 91.91% of the women in our sample with downward mobility have gone from a medium to a low SES, the results seem to indicate that the process of social mobility leads to the incorporation of lifestyle habits from the achieved SES, coinciding with the results of another study [33].

Most published studies observe that men and women with a low SES are less physically active, regardless of age [34–36]. This is in line with our results for younger women and men, but not for older men. This may be due to the fact that occupation is acting as a confounder. Manual occupations such as masonry or agriculture are typical of the population with a low educational level, which implies physical activity and, possibly, later retirement [37].

Regarding the co-occurrence of behaviours, studies have reported that men accumulate more risk behaviours than women [9, 11, 38], in line with our results showing that women accumulate a greater number of healthy behaviours. These differences can again be explained by the impact of traditional gender roles, which influence men and women’s different conception of health and, therefore, the related behaviours [39]. Traditionally speaking, women’s behaviours are influenced by the role of the female caregiver, which predisposes them to take care of their health and that of their relatives while men tend to have fewer healthy behaviours due to the influence of masculine stereotypes [40].

Inequalities in the co-occurrence of healthy behaviours were observed in both genders and age groups by origin and destination SES and social mobility. Older women are more likely to have co-occurrence if they have a low SES, independently of it is origin or destination SES. This could be explained by the fact that, as observed in the analysis of each healthy behaviour, older women with a low SES are more likely to be non-smokers and consume moderate amounts of alcohol, which may be due to the impact traditional gender roles have had on older women.

In the case of men, the same behaviour pattern is observed in those with a low origin SES as those with upward mobility, regardless of age. These results once again confirm the socialization hypothesis [17].

This study provides an approach to the analysis of the impact of origin and destination SES and social mobility on healthy behaviours due to the intersection of age and gender. The results reinforce the idea that health and health behaviours are socially determined and strongly linked to the specific values, norms and social resources of each social strata. The study on social mobility has provided a complex vision of the influence of origin and destination SES on the assumption of healthy behaviours. It has shown that in men, healthy behaviours from the low social position of origin are maintained when there is social mobility.

One of the limitations of this study could be the social desirability bias, meaning that participants respond according to what is considered socially correct or expected, overestimating healthy behaviours in all social strata. However, the fact that the questionnaire is administered by qualified interviewers who are continuously learning and that the FFQ questionnaire was validated in Spain reduces this type of bias [19, 41].

On the other hand, an effect of reverse causality could be present, common in studies with a cross-sectional design. Taking other studies [14] into account, the association between health and SES is bidirectional, since healthy behaviours could also influence the population’s social mobility.

It is important to take into account the possible confounding factors behind SES and healthy behaviours. In this article, only the confounding factors of sex, age and region are controlled through adjustment or stratification, but other possible confounding factors could affect our results (such as occupation, marital status, Body Mass Index etc.). Despite this, they provide an approximation of how origin and destination SES and social mobility impact healthy behaviours.

Despite the fact that the sample was randomly selected in each of the regions taking part in the study and that this aspect reduced selection bias, age may be represented unequally between men and women. To overcome this limitation, the sample was stratified by age and sex.

Although the results obtained coincide with the findings of recently published studies on this topic, it would be advisable to replicate this study in younger cohorts.

## Conclusion

This study shows that the impact of origin and destination SES and social mobility on the prevalence and co-occurrence of healthy behaviours differs by gender and age. These differences justify the importance of pursing a health-focused approach in all policies based on the principle of health promotion [42, 43], as well as interventions adapted to the needs of each social group in order to improve equity in health [44].
